# Determining the level of stromal and epithelial cells activity in normal and hyperplastic endometrium of late reproductive and perimenopausal women

**DOI:** 10.25122/jml-2022-0305

**Published:** 2023-02

**Authors:** Zinaida Vasilyvna Chumak, Volodymyr Victorovich Artyomenko, Mykola Vitaliiovich Shapoval, Liudmyla Volodymyrivna Mnih, Ganna Volodymyrivna Kozhukhar, Serhii Vasilyovich Derishov

**Affiliations:** 1 Odessa City Center for Climacteric Problems, Odesa, Ukraine; 2 Department of Obstetrics and Gynecology, Odesa National Medical University, Odesa, Ukraine

**Keywords:** hyperplastic endometrial processes, p53, BCL-2, carcinogenesis, apoptosis, perimenopausal period, late reproductive period

## Abstract

Hyperplastic processes of the endometrium (HPE) are a group of benign endometrial and stromal cells that have undergone altered growth. This study aimed to investigate the potential role of hypoxia (as indicated by Hif-1α) and apoptosis markers (p53 and BCL-2) in the development of hyperplastic processes of the endometrium (HPE). Results showed that endometrial cells with atypical hyperplasia had increased levels of Hif-1ɑ, which indicates the presence of endometrial hypoxia and may trigger pathological manifestations. Though this result was not statistically significant, it could be the cause of atypia hyperplasia in the late reproductive period (Hif-1ɑ=1.89±0.09 units) and the perimenopausal period (Hif-1ɑ=2.09±0.07 units). Additionally, the study found that p53 markers were elevated in epithelial cells in the late reproductive period, and similar patterns were observed in the perimenopausal period, with the biggest expression in atypical hyperplasia. The study also found that the high expression of BCL-2 indicator (+++) was less common in late reproductive period women with atypia than those without it (χ^2^=7.2 p=0.01). A similar situation was observed in women in the perimenopausal period (χ^2^=4.2 p=0.04). These findings suggest that hypoxia may play a role in the development of HPE, as well as changes in apoptotic markers present in the endometrial tissue.

## INTRODUCTION

Hyperplastic processes of the endometrium (HPE) are a group of benign altered endometrial and stromal cells that can be influenced by various heterogeneous factors [1, 2]. The main molecular genetic mechanisms that lead to the development of HPE are complex and not fully understood. However, research has shown that it is associated with unbalanced estrogens, inadequate progesterone, and other molecular, immunohistochemical, and genetic changes in the endometrial tissue [3–7]. In some cases, these factors can lead to malignant changes in human organisms [2, 7].

In the scientific literature, the molecular genetic carcinogenesis theory, which is based on chemical, radiation, and viral tumor origin theory, has been put forward [4, 8]. The emerging tumor has to a certain extent, the self-sustaining mutagenic stimuli qualities together with constant division and insensitivity to antimitogenic signals; it has unlimited replicative potential, a reduced ability to undergo apoptosis, a tendency to tissue invasion and metastasis, and other characteristic factors of tissue changes [6–8].

In the global scientific literature, considerable importance is attached to mechanisms and processes underlying hypoxia in the tissue. Hypoxia has been found to play a role in the development and progression of malignant cells, as first proposed by O. Warburg in 1924. Various regulatory factors are believed to be involved in the tissue-level response to decreased oxygen levels. One such factor is Hif-1ɑ, whose activity increases when the oxygen concentration in the blood decreases. This marker is suitable for tissue adaptation to hypoxia, and many researchers have studied its concentration in tumor cells. Research is ongoing to understand the influence mechanisms on Hif-1ɑ and to develop therapeutic measures to target the malignancy processes or their development [4, 5, 7].

Apoptosis and proliferation support cell homeostasis in the body. While some studies have found changes in these processes in hyperplastic conditions, especially in atypical endometrial hyperplasia [1, 4], other studies have not been able to replicate these findings [2, 5]. However, not all cases of atypical endometrial hyperplasia will progress to cancer [3, 6].

Examination of DNA damage often reveals mutations in the TP53 gene, which can lead to the production of a non-functional p53 protein. This protein is present in about half of all cancer cells, and its mutations are considered an early event in cancer development [1, 4]. However, it is important to note that the presence of p53 mutations alone is not the only factor in the development of malignancy, and other markers also play a role in characterizing the specific characteristics and presence of a tumor [4, 5, 7].

The cell division process is a complex system that involves multiple mechanisms for maintaining genetic stability and controlling cell growth. When certain mutations occur, careful genetic state control is necessary. If there are changes, proliferation is temporarily stopped, and if the damage cannot be reversed, the cell may undergo programmed cell death. Protein p53 plays a significant role in this process, but apoptosis antagonists are also important [6–8].

The BCL-2 protein, a product of the Bcl-2 gene and a cell survival factor, protects cells from apoptosis processes. However, studies have shown that high levels of BCL-2 protein expression may have oncogenic properties and have been observed in atypical endometrial hyperplasia [4, 6, 8]. Other researchers found a decreased expression of BCL-2 in atypical hyperplasia and endometrial adenocarcinoma [1, 3], highlighting the need for further research to understand the specific mechanisms and roles of BCL-2 in oncogenesis and potential therapeutic interventions [2, 7].

HPEs represent a developing risk for oncological pathology, with 30–70% of atypical processes progressing to cancer within 1–3 years [1, 7]. Moreover, recent studies have highlighted the potential link between HPEs in late reproductive-age women and the development of conditions such as hypomenstrual syndrome, obesity, and polycystic ovarian syndrome in their daughters [9, 10].

This study aimed to determine, compare and investigate the correlation of Hif-1ɑ, p53, and BCL-2 markers in the stromal and epithelial cells of normal, hyperplastic, and atypical endometrium in women of different ages.

## MATERIAL AND METHODS

The expression of Hif-1ɑ, p53, and BCL-2 markers was studied in 183 endometrial tissue samples from women in the late reproductive (35–44 years) and perimenopausal (45–54) periods divided into 6 groups. 2 control groups were established: one for late reproductive age women (Group I) and second for perimenopausal women (Group IV) to fulfill all the criteria of comparison. Group I included 32 patients without pathological changes in the endometrium, group II included 36 late reproductive-age women with hyperplastic processes without atypia, group III consisted of 22 late reproductive-age patients with atypical endometrial hyperplasia, group IV included 30 perimenopausal-age women without proliferative tissue changes, group V included 38 perimenopausal women without atypical hyperplasia, and group VI included 25 perimenopausal age patients with atypical hyperplasia.

For immunohistochemical examination, the material obtained during scraping was fixed in 10% neutral buffered formalin and embedded in paraffin blocks. Antibodies to BCL-2, p53 (DAKO – Germany) were used as primary specific antibodies. P53 expression and evaluation were carried out by calculating the stained nuclei per 100 cells percentage in the glands' epithelium and in the stroma. The expression of BCL-2 was evaluated based on the brown color intensity, with the staining intensity being evaluated by points as follows: no staining – (0), weak staining – (+), moderate staining – (++), strong staining – (+++). The color intensity was evaluated in points, and the H-score was noted. Hif-1α expression was performed at the mRNA level by cDNA polymerase chain reaction. Statistical processing was performed using the dispersion method and correlation analysis using standard MS Excel macros (Microsoft Inc., USA).

## RESULTS

We evaluated the expression of Hif-1ɑ in normal, hyperplastic, and atypical endometrium of women in late reproductive and perimenopausal stages.

**Figure 1:**
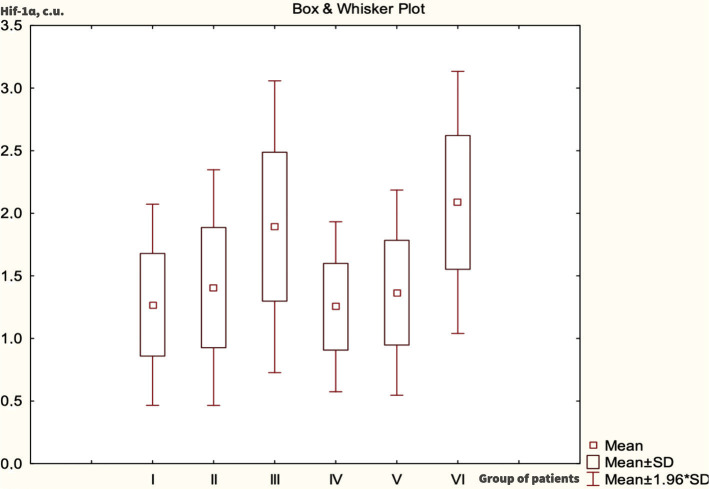
The expression of Hif-1ɑ in endometrial tissue cells (c.u.).

In Figure 1, we present changes in Hif-1ɑ expression in endometrial tissue under different endometrial conditions. These changes in the endometrial tissue during the pathological processes studied in different age categories are very important.

Our study found that while the levels of Hif-1α were not statistically significant, the decreased oxygen concentration in endometrial tissue is a key characteristic of Hif-1α expression. This is particularly relevant in the presence of atypical processes, which is essential for our research. During the late reproductive period, Hif-1ɑ was 1.89±0.09 c.u., and in the perimenopausal period, it was 2.09±0.07 c.u. These variations can influence the development of pathological manifestations at the tissue level, especially in cases of hyperproliferation.

Our research clearly shows that decreased oxygen concentration in endometrial tissue may be a primary factor in the development of hyperproliferation and atypization in endometrial cells, which in turn can affect the levels of p53, a protein that regulates cell proliferation, and BCL-2, a protein that regulates cell apoptosis.

Taking into account the importance of certain molecular genetic factors in the development and progression of proliferative process in the endometrial tissue, we evaluated p53, BCL-2, and Hif-1ɑ markers in relation to patients' age and aimed to establish their possible prognostic value for the development of HPE.

The study of p53 indicators and their changes in endometrial tissue during the pathological process are presented in Table 1.

**Table 1:** Expression of p53 in epithelial and stromal cells of endometrial tissue.

Group	Epithelial cells			Stromal cells		
	%	M	SE	%	M	SE
**I**	2.12	2.05	0.03	-	-	-
**II**	7.04	2.58	0.05	1.65	2.02	0.02
**III**	35.52	7.99*	0.58	19.88	6.33	2.22
**IV**	2.33	2.04	0.03	0.94	2.04	0.02
**V**	9.74	6.78** ^#^	1.03	1.56	2.01	0.06
**VI**	43.44	6.33**	3.67	18.67	5.53	2.43

* – differences with the control group I were statistically significant (p<0.05); ** – differences with the control group IV were statistically significant (p<0.05); ^#^ – differences between stroma and epithelium cells were statistically significant (p<0.05).

We analyzed the expression of the p53 marker (Table 1) in epithelial and stromal cells of endometrial tissue. There were statistical changes in the expression of p53 in the group of late reproductive women with normal endometrium. However, there were no expression factors detected in stromal cells, indicating that such changes may represent an inactive process. Subsequently, the timing of tissue collection and processing is crucial when conducting research and determining prognostic value, as it can affect the accuracy of the results. In our sample, the expression of p53 was 2.12% (2.05±0.03) in epithelial cells and was absent in stromal cells. In the perimenopausal period, these changes were characterized by insignificant indicators. However, we established a correlation between changes in p53 expression and the dependence and possibility of proliferation in the endometrial tissue. This was particularly evident in atypical hyperplasia, where there was an increase in the expression of p53 in epithelial cells during the late reproductive period (35.52%; 7.99±0.58) and endometrial tissue stroma cells (19.88%; 6.33±2.22), which significantly outweighs similar indicators in HPE and the case of unchanged proliferative processes. A similar pattern was found in atypical endometrial hyperplasia in the perimenopausal period (p53=43.44%; 6.33±3.67) in the endometrial epithelium cells and p53=18.67%; 5.53±2, 43 in stroma cells, respectively.

These indicators suggest that atypical endometrial hyperplasia may affect the endometrial tissue proliferation process and may be accompanied by specific changes that can potentially lead to malignancy in the presence of other factors. However, it is important to note that this process does not always occur, as other factors may promote or inhibit this system.

A more detailed analysis of the p53 marker indicators in the perimenopausal period revealed a significant difference in expression between the data at HPE. Specifically, the expression of p53 in epithelial cells was 9.74% (±1.03), and in stromal cells, it was 1.56% (±0.06) which was significantly different from both the control and indicators in stromal cells (p<0.05). These findings suggest that certain factors may influence the endometrial tissue's condition during the perimenopausal period and lead to the development of hyperplastic changes. These changes may be only associated with changes at the tissue level, and further research is needed to understand the underlying mechanisms.

The differences between the groups are near the limit of statistical significance; therefore, to further clarify their potential role in predicting the course of hyperproliferative states, we conducted additional analysis of markers that affect and inhibit proliferative processes. We determined and analyzed the BCL-2 apoptosis inhibition index (Table 2).

**Table 2:** BCL-2 expression in epithelial and stromal cells in endometrial tissue.

Group	Epithelial cells			Stromal cells		
	%	M	SE	%	M	SE
**I**	10.24	16.96*	0.9	2.04	2.0	0.03
**II**	12.33	14.04*	1.2	1.75	2.0	0.2
**III**	10.42	11.97*	1.3	2.07	2.8	0.03
**IV**	11.01	14.03*	1.1	3.87	3.0	0.6
**V**	9.39	11.02*	1.1	4.84	4.0	0.03
**VI**	13.98	16.01*	1.0	3.96	3.0	0.2

* – differences between stromal and epithelial cells were statistically significant (p<0.05).

In the analysis of BCL-2 expression in relation to age-related changes in endometrial tissue, characteristic disturbances were found in epithelial and stromal cells. These changes were statistically significant in terms of the presence of the marker in epithelial and stromal cells since this marker clearly progresses in epithelial cells and is corrected in time in the stromal cells, which prevents and controls proliferative changes (p<0.05). Such changes confirm the presence of immunohistochemical disorders in both epithelial and stromal cells, but the stroma can correct these pathological changes.

From our analysis of BCL-2 expression and its expression degree, we established that changes occur in both epithelial and stromal cells. However, the changes in epithelial cells were more significant than in stromal cells, where they are almost not pronounced, or slight fluctuations in the BCL-2 marker expression are manifested. These corrections are particularly important during the perimenopausal period, which should be taken into account for systemic analysis, as not only hormonal but also other metabolic changes are manifested in a woman's body, which can affect the patient's general condition and potential malignancy.

The BCL-2 apoptosis inhibitor indicators increase with the strengthening of hyperproliferation processes, which is manifested in an atypical condition, but not significantly enough. Further analysis of BCL-2 expression, including its accumulation capacity, is presented in Table 3.

**Table 3:** BCL-2 expression in epithelial cells of endometrial tissue.

Marker	Study groups											
	I		II		III		IV		V		VI	
	Abs.	%	Abs.	%	Abs.	%	Abs.	%	Abs.	%	Abs.	%
**BCL-2 (+++)**	14	43.75	9	25.00	3*	13.64	13	43.33	10	26.32	2**	8.00
**BCL-2 (++)**	16	50.00	21	58.33	9	40.91	15	50.00	22	57.89	5**	20.00
**BCL-2 (+)**	2	6.25	6	16.67	7*	31.82	2	6.67	5	13.16	10	60.00
**BCL-2 (0)**	0	0	0	0	3	13.64	0	0	1	2.63	3	12.00

* – differences with the control group I were statistically significant (p<0.05); ** – differences with the control group IV were statistically significant (p<0.05).

When comparing the frequency of high expression detection of BCL-2 indicator (+++), we found that this variant occurs much less often in group III than in group I (χ^2^=7.2, p=0.01). A similar pattern was observed in the endometrial tissue among women in group VI (χ^2^=4.2, p=0.04). In terms of weakly positive BCL-2 indicator expression (0), we found that there were significantly more such women in group III (χ^2^=4.4, p=0.04) and group VI (χ^2^=4.7, p=0.04) when compared to the control group.

Qualitative and quantitative changes in the indicator expression have a cumulative nature, so it is informative to analyze this indicator in a complex manner when investigating the possibility of malignancy in endometrial tissue cells. Our research showed a difference in the occurrence of BCL-2 expression in cells with endometrial disruption, such as HPE or atypical hyperplasia. Changes in endometrium epithelial cells in glandular and atypical hyperplasia are characterized by a decrease in apoptosis inhibition processes, particularly in atypical hyperplasia, where during the late reproductive period, it was 13.64%, and in the perimenopausal period, it was 12.00%.

## DISCUSSION

According to prognostic assessments in Ukraine, the incidence rate of HPE may increase by 30–95% by 2050. This is particularly concerning for breast, cervical and uterine cancer, which can significantly affect the demographic situation in Ukraine. The growth and development of these pathological conditions is not only a medical issue but also a social problem [1, 2].

Hyperplastic endometrial processes are characterized by abnormal growth and changes in the glandular and stromal cells. Some researchers have found that these changes can be accompanied by atypical hyperplasia in the epithelial cells as weakly positive and characterized by similar changes in the tissue. The prevalence of these changes corresponded to 58.33% in late reproductive-age women, and 57.89% in perimenopausal women, indicating the presence of cellular and tissue disorders in HPE [4, 6]. It is necessary to consider that taking endometrial tissue samples was not always associated with menstrual cycle phases, and sometimes, medical assistance was provided after sampling. In our work, the apoptosis proteins inhibitor – BCL-2 was more actively expressed at the cellular atypia appearance, which may indicate their importance in the atypical change processes and possible changes in malignancy.

Evidence suggests that increased BCL-2 protein expression may play a role in the early stages of hyperplastic processes in the endometrial tissue and allows cells to survive during hyperproliferation. Our data could confirm the decrease in apoptosis in conditions that contribute to malignancy in the endometrial tissue.

Malignant processes also increase the synthesis of BCL-2 protein, an apoptosis inhibitor. Mutations in the p53 protein make cells insensitive to proliferation and apoptosis signals. Only the combination of data on the status of this trigger can provide insight into the potential of the cell. While many studies focus on the individual markers associated with oncological processes, understanding the complex interactions between these markers is crucial in understanding the violations that occur.

The subject of HPE development and the identification of adenocarcinoma remains an ongoing area of scientific, clinical, and research interest. The success of treatment and the prevention of HPE development remain relevant in many medical research areas and cannot be achieved without reaching an understanding and indicative values in fundamental science. The development of modern molecular biology methods provides a clearer understanding of new aspects of carcinogenesis and the influence and existence of certain tumor development factors. It is quite promising, especially in medical practice, to identify certain signs that direct the tumor process development and possible options for its correction.

We consider characteristic hypoxic changes in the endometrium to be related mainly to tissue level changes in the development of different types of HPE in this age period, as they contribute to future mortality in this group of women.

Determining the direction of the endometrial process is possible by diagnosing it at the initial stages of proliferation, which may indicate its biological aggressiveness and the specifics of its biological process in the future. For researchers, it is necessary to consider the endometrial cell parameters, evaluate the Hif-1ɑ factors, and the stages of apoptosis p53, BCL-2.

Our study determined the expression of Hif-1ɑ, p53, and BCL-2 markers depending on the woman's endometrial tissue state and age. Under certain conditions, it was promising to investigate the hypoxia manifestations in tissue characterized by Hif-1ɑ and then progressive changes in p53 and BCL-2 expression. Since a woman's body is a fairly significant compensatory system, the complexity of these techniques can influence the process development.

Several studies revealed that the molecular mechanisms of oncological transformation and tumor progression are general, and certain active marker parameters could be of great importance for future findings in the fight against malignancy.

## CONCLUSIONS

The development of proliferative changes in the endometrium is characterized by a hypoxic state, as evidenced by the increased expression of the Hif-1ɑ marker. This information can be used to improve the condition and particularly target disturbances in hyperproliferative processes and atypical cells, which may indicate a risk of malignancy. The expression of the p53 marker is also promising, but the growth patterns in both epithelial and stromal cells should be further evaluated. Additionally, the increased expression of the apoptosis inhibitor BCL-2 in epithelial and stromal cells can provide insight into the progression of proliferative processes.

Overall, molecular genetic and immunohistochemical markers play a crucial role in determining the effectiveness of treatment for cancer patients, as they provide insight into the progression and severity of the disease.

## Data Availability

Further data is available from the corresponding author on reasonable request.

## Personal thanks

Our team would like to thank LLC Artemis LTD (Ukraine) for their support in manuscript interpretation.
